# Exosomes as Drug Carriers in Anti-Cancer Therapy

**DOI:** 10.3389/fcell.2022.728616

**Published:** 2022-01-26

**Authors:** Lan Chen, Li Wang, Lingling Zhu, Zihan Xu, Yanyang Liu, Zhixi Li, Jin Zhou, Feng Luo

**Affiliations:** ^1^ Lung Cancer Center, Cancer Center, State Key Laboratory of Biotherapy, West China Hospital of Sichuan University, Chengdu, China; ^2^ School of Medicine, Sichuan Cancer Hospital and Institute, Sichuan Cancer Center, University of Electronic Science and Technology of China, Chengdu, China

**Keywords:** exosomes, drug delivery, cancer therapy, targetability, engineering

## Abstract

Over the years, there has been a high demand for developing new safe and effective drug carriers for cancer therapy. Emerging studies have indicated that exosomes can serve as potent therapeutic carriers since they offer low immunogenicity, high stability, innate and acquired targetability, and the stimulation of anti-cancer immune responses. Yet, the development of exosome-based drug delivery systems remains challenging due to their heterogeneity, low yield, and limited drug loading efficiency. Herein, we summarized the current application of exosomes derived from different cells as drug carriers in anti-cancer therapy *in vitro* and *in vivo*. We also discussed the challenges and prospects of exosome-based drug delivery systems in cancer therapy.

## 1 Introduction

Cancer is a major public health problem and a leading cause of death worldwide. In 2020, 19.3 million new cancer cases and approximately 10.0 million cancer-related deaths were reported (Sung et al., 2021). Surgery is still considered the golden approach for patients with early-stage cancer, while chemotherapy, radiotherapy, and targeted drug therapy are commonly used to treat advanced-stage cancer patients. Yet, these therapies have certain limitations. For example, conventional chemotherapy and radiotherapy lead to side effects such as vomiting reaction, myelosuppression, radiation dermatitis, and radiation pneumonitis. Other limitations of chemotherapy are poor bioavailability, high-dose requirements, low therapeutic indices, development of multiple drug resistance, and non-specific targeting. On the other hand, targeted drugs such as gefitinib, osimertinib, and sorafenib have been shown to have high selectivity and low cytotoxicity; however, they may lead to drug resistance over time. Hence, the exploration of new and revolutionary therapeutic agents is urgently needed.

Recently, immunotherapy has become very popular in the treatment of cancer therapy. Immune checkpoint blockade (ICB), such as antibodies against programmed death receptor (PD)-1, PD ligand (PD-L)-1, and cytotoxic T-lymphocyte antigen 4 (CTLA-4), has shown to be very promising in treating cancer patients. However, immune checkpoint inhibitors fail to control tumor progression in many patients due to the immunosuppressive microenvironment in tumor tissues induced by cancer cells, immune cells, and tumor-associated stromal cells (Chabanon et al., 2016; Maleki Vareki et al., 2017). Thus, new anti-cancer therapeutic strategies should increase targetability, overcome drug resistance and/or improve immunosuppressive tumor microenvironment.

Over the past decade, numerous synthetic drug carriers, such as liposomes and nanoparticles, have been developed to cure cancer (Perez-Herrero and Fernandez-Medarde, 2015). These carries can either passively or actively target cancerous cells, reducing adverse side effects and improving therapeutic efficacy. For example, Ghassami *et al.* designed polymeric nanoparticles combined with an aptamer specifically recognizing human epidermal growth factor receptor 2 (Her 2), which could directly deliver docetaxel to cancer cells ovexxpressing Her 2 *in vitro* and *in vivo* (Kronschnabl et al., 2020). In addition, Eskiler *et al.* demonstrated that, compared to free talazoparib, encapsulating talazoparib with solid lipid nanoparticles could enhance the stability and bioavailability of talazoparib, thereby increasing the cytotoxic effect to multidrug-resistant breast cancer cells (Pu et al., 2019). Moreover, Xiang *et al.* showed that melittin-lipid nanoparticles promote tumor antigen release *in situ* and activate antigen-presenting cells in lymph nodes, leading to a 3.6-fold enhancement of antigen-specific CD8^+^T cell responses (Yu et al., 2020).

Despite the aforementioned advantages, liposomes and nanoparticles still have a few shortcomings, which limit their application in clinical practice. For example, overcoming the reticuloendothelial system has long been a vital challenge to liposomes and nanoparticles as drug carriers (Tang et al., 2019). Additionally, biological barriers reduce their bioavailability and limit therapeutic efficacy (Arrighetti et al., 2019). In addition, nanoparticles have been associated with certain adverse effects *in vivo*, such as cardiovascular toxicity, mitochondrial dysfunction, and platelet aggregation (De Jong and Borm, 2008).

Exosomes are natural nanoscale vesicles secreted by almost all living cells. They are cell-derived membranous structures capable of transporting various active biomolecules from host cells to recipient cells. Recently, exosomes have been proposed as new biological drug carriers. Compared to liposomes and nanoparticles, exosomes have the following advantages: 1) low immunogenicity; 2) low clearance of reticuloendothelial system *in vivo*; 3) higher bioavailability (can easily pass through biological barriers, including intestinal barrier, blood-brain barrier, and placental barrier); 4) low accumulative toxicity in normal tissues; 5) selectively delivering anti-cancer drugs into cancer cells via ligand-receptor interaction or endocytosis to overcome drug resistance mediated by P-glycoprotein or other multidrug resistance-associated proteins (Kim et al., 2016); 6) the plasticity of acquired targetability to cancer cells; 7) the stimulation of anti-tumor immune responses.

This review discussed the application, challenges, and prospects of exosome-based drug delivery systems in cancer therapy.

## 2 Characteristics and Function of Exosomes

### 2.1 Exosomes Characteristics

**FIGURE 1:**
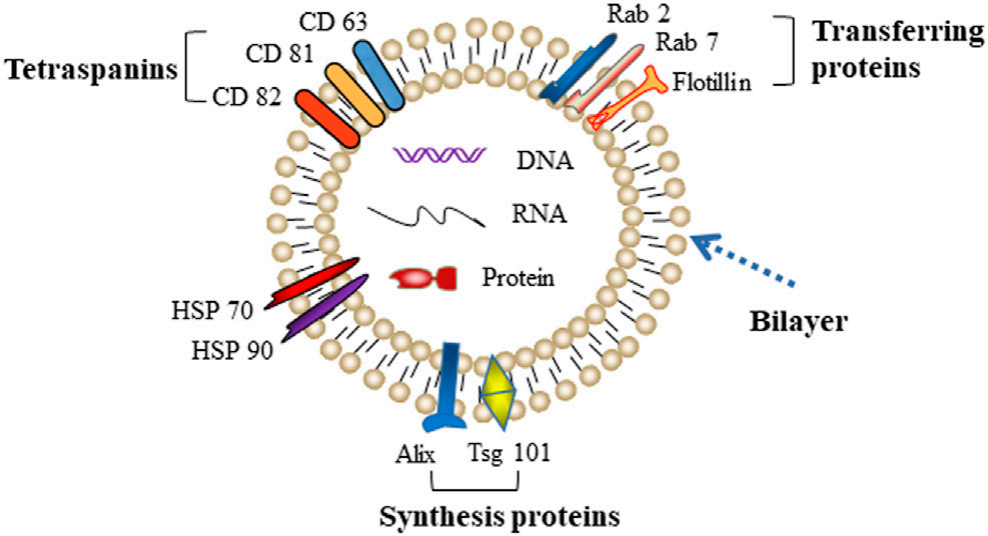
Typical characteristic of exosomes.

Exosomes were first reported by Johnstone in 1987, who discovered a vesicle formation during the maturation process of reticulocytes (Harding et al., 1983; Pan et al., 1985). A few years later, several other groups revealed that the biogenesis of exosomes begins with the internal budding of endosomes, which then forms intraluminal vesicles (ILVs) and further matures into late endosomes, namely multivesicular bodies (MVBs). MVBs eventually merge with the plasma membrane and release ILVs into the extracellular space as exosomes. Exosomes are typical “cup-like” structure vesicles with a diameter of 30–150 nm. The main molecular components of exosomes are cell-derived lipids, glycoconjugates, proteins, and nucleic acids (lncRNA, microRNA, and DNA). Due to an evolutionarily conserved set of proteins present in exosomes, such as fusion and transferring proteins (Rab2, Rab7, flotillin, and annexin), heat shock proteins (HSP70, HSP90), integrins, tetraspanins (CD63, CD81, and CD82), cytoskeleton proteins (β-actin and tubulin), synthesis proteins (Alix and Tsg101), part of which are considered as special markers used for identification of exosomes (Figure 1) (Mashouri et al., 2019).

### 2.2 Functions of Exosomes

Exosomes have pivotal functions in intercellular communication through delivering their internal components, such as mRNAs, miRNAs, and proteins. They regulate various normal physiological activities and may participate in the initiation and progression of tumors. Sun *et al.* demonstrated that glioblastoma stem cell-derived exosomes enhance the self-renewal ability of glioma cells *in vitro* and *in vivo* (Sun et al., 2020). Moreover, Hu *et al.* revealed that cancer-associated fibroblasts secrete exosomes, which promote metastasis and chemoresistance of colorectal cancer (Hu et al., 2019). In addition, our team also revealed that lung cancer stem cell-derived exosomes promoted the migration and invasion of lung cancer cells (Wang et al., 2020). Considering that exosomes are packed with different proteins and RNAs, recent studies suggested that exosomes could be used as reliable clinical diagnostic and prognostic biomarkers for certain diseases, including cancer. For instance, the HOXA transcript at the distal tip (HOTTIP) is a long noncoding RNA (lncRNA) involved in the proliferation of various cancer cells. Zhao *et al.* found that exosomal HOTTIP could be utilized as a potential biomarker for the diagnosis of gastric cancer, and its level was an independent factor for poor prognosis in patients with gastric cancer (Zhao et al., 2018). In addition, high levels of exosomal miR-10b-5p, miR-23b-3p, and miR-21-5p are significant biomarkers of poor prognosis in patients with lung cancer (Liu et al., 2017). Moreover, plasma-derived exosomes, which are rich in membrane-bound protein New York-esophageal-1, were found to be closely associated with shorter overall survival of patients with non-small cell lung cancer (NSCLC) (Sandfeld-Paulsen et al., 2016).

### 3 Exosome-Based Drug Delivery Systems

**FIGURE 2:**
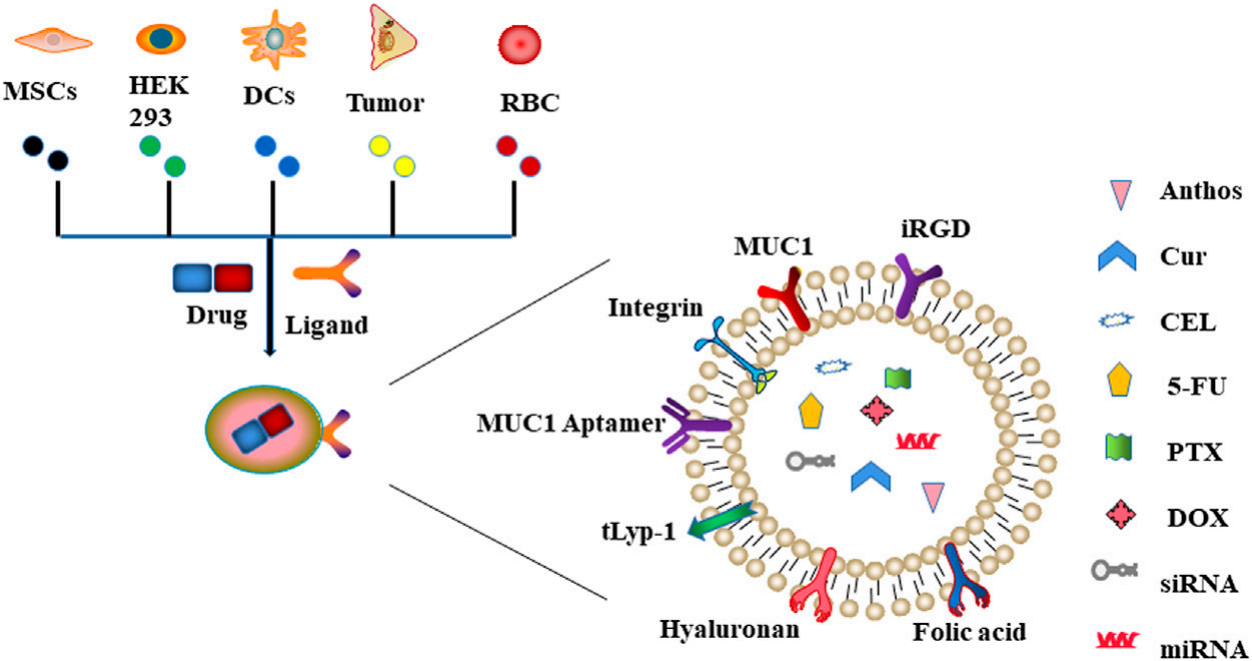
Schematic representation of exosome-based drug delivery systems. Exosomes derived from different types of cells (including MSCs, HEK293, DCs, tumor, and RBC) can deliver drugs to cancer cells through innate or acquired ligands expressed on the surface of exosomes.

Through passive or active targeting, exosomes can deliver therapeutic agents such as nucleic acids, proteins, and small molecule drugs to cancer cells (Zhang and Yu, 2019). This potential can be maximized by utilizing exosomes as natural nanoplatforms for drug delivery. Herein, we summarized the most common exosome-based drug delivery systems (Figure 2).

### 3.1 Milk-Derived Exosomes

Milk is a good source of many essential nutrients such as protein, calcium, and vitamin D, and a vital part of a balanced diet. In 2007, exosomes were successfully isolated from human milk for the first time. So far, exosomes have been successively identified from cow, pig, kangaroo, camel, rat, horse, panda, yak, sheep, and goat milk (Sedykh et al., 2020). Compared with exosomes from other origins, milk-derived exosomes are easily accessible, have high yield, and are non-cytotoxic, which makes them a suitable natural source for the production of exosomes.

#### 3.1.1 Role of Milk-Derived Exosomes as Drug Carriers

Badawy AA *et al.* first reported that milk-derived exosomes exert a moderate inhibitory effect on the proliferation of breast cancer (Badawy et al., 2018). At that time, Radha *et al.* have further demonstrated that milk-derived exosomes loaded with anti-cancer drugs (anthocyanins, withaferin A, curcumin, paclitaxel (PTX), and docetaxel, respectively) exerted stronger proliferation-inhibiting effects on lung and breast cancer compared with the free drugs (Munagala et al., 2016). Moreover, milk-derived exosomes are evolutionally conserved and maintain the integrity of their contents when passing through the gastrointestinal tract; thus, they may be used orally, avoiding patient intolerance caused by the intravenous injection of anti-cancer drugs. Jamie *et al.* discovered that milk-derived exosomes are well tolerated in the gastrointestinal tract’s acidic environment and can absorb by the gastrointestinal epithelium via the “neonatal” Fc receptor (Betker et al., 2019). In a recent preclinical study, mice were administered with milk-derived exosomes labeled with fluorescent dye DIR via oral gavage. A strong fluorescent signal was then seen in the blood 30 min after gavage and remained relatively constant after 60–240 min, which further suggested that milk-derived exosomes can translocate from the gut to the blood in mice. In addition, Sergey *et al.* demonstrated that, after encapsulated curcumin in milk-derived exosomes, the stability and water solubility of curcumin in the acidic intestinal environment were improved, which increased curcumin absorption into blood across the intestinal epithelium (Vashisht et al., 2017). In addition, Farrukh *et al.* found that oral administration of milk-derived exosomes loaded with celastrol (CEL) could increase drug stability and cellular uptake ability, and in turn, reduce tumor growth in lung cancer-bearing mice compared with the free CEL (Aqil et al., 2016). Natural berry anthocyanidins (Anthos) have high activity but are also susceptible to environmental factors such as pH and temperature, exhibiting poor oral bioavailability and stability. Farrukh *et al.* found that modified milk-derived exosomes loaded with Anthos could increase the stability and bioavailability of Anthos and greatly improve the anti-cancer efficacy against ovarian cancer (Aqil et al., 2017).

RNA interference (RNAi) refers to the process of homologous mRNA-specific silencing induced by exogenous or endogenous double-stranded RNA. Compared to the small molecules mentioned above, RNAi exerts more prominent anti-cancer effects by selectively inhibiting pivotal genes controlling proliferation, migration, invasion, differentiation, or other malignant biological behaviors of cancer cells. Gene therapy through RNAi showed targeted advantage and few adverse reactions *in vitro* and *in vivo*. However, evidence regarding its clinical application is limited due to the obvious off-target effects of siRNA and the instability of siRNA delivery *in vivo*. Farrukh *et al.* (Aqil et al., 2019) loaded Texas Red–labeled siRNA into milk-derived exosomes and then co-cultured these exosomes with lung cancer cells. They demonstrated that milk-derived exosomes could deliver siRNA to lung cancer cells. Furthermore, Hong *et al.* verified that milk-derived exosomes carrying Bcl-2-siRNA could efficiently penetrate the cell membrane and suppress the invasion and migration of pancreatic cancer cells by downregulating matrix metalloproteinases 2 (MMP2), MMP9, vimentin, and N-cadherin (Tao et al., 2020). However, further evidence is needed to support the anti-cancer effects of milk-derived exosomes carrying siRNA *in vivo*.

#### 3.1.2 Shortcomings of Milk-Derived Exosomes as Drug Carriers

Though the effect of tumor suppression for oral administration of milk-derived exosomes carrying anti-cancer drugs has been verified *in vivo* and *in vitro*, the tissue distribution of these exosomes *in vivo* needs to be further investigated. Radha *et al.* found that, after administration of DIR-labelled milk-derived exosomes by oral gavage in mice, the biodistribution of these exosomes within the liver, lung, kidney, spleen, and brain was uniform (Munagala et al., 2016). Moreover, after administrating radioactively ^99^mT c labeled milk-derived exosomes by intravenous route in healthy mice, Gonzalez *et al.* (Gonzalez et al., 2020) discovered that the radiation signal accumulation was the highest in the liver and spleen followed by stomach and kidneys.

Based on the non-specificity of tissue distribution of milk-derived exosomes *in vivo*, targeted drug delivery system was paid much attention in recent years. Several studies showed that milk-derived exosomes could be directly modified by hyaluronan or folic acid (FA), thus enhancing tumor targetability. Dan *et al.* co-incubated CD44 specific binding ligand hyaluronan with milk-derived exosomes to deliver doxorubicin (DOX) and found that these exosomes could target CD44 positive cancer cells to induce apoptosis (Li D. et al., 2020). Moreover, Raghuram *et al.* discovered that milk-derived exosomes loaded with paclitaxel and then modified with FA could especially target tumor tissues and significantly suppress lung cancer growth in mice via oral administration (Kandimalla et al., 2021). Moreover, Farrukh *et al.* (Aqil et al., 2019) found that FA modified milk-derived exosomes loaded with siRNA-KRAS could significantly accumulate in tumor tissues of lung cancer-bearing mice via intravenous route and obviously inhibit tumor growth.

However, receptors of hyaluronan or FA are also endogenously expressed in healthy cells/tissues. Wang *et al.* reported that the receptors of hyaluronan are ubiquitously present in many other cells, including fibroblasts, epithelial cells, endothelial cells, and smooth muscle cells (Suleiman et al., 2018). Also, Nikki *et al.* demonstrated that the expression of folate receptors had a widespread distribution in normal tissues such as the kidney, lung, intestine, heart, and liver (Parker et al., 2005). Therefore, the question remains whether this method is safe enough.

### 3.2 Mesenchymal Stem Cell-Derived Exosomes

Mesenchymal stem cells (MSCs) are cells isolated from multiple biological tissues such as adult bone marrow, adipose tissues, placenta, and umbilical cord. *In vitro*, MSCs have high proliferation ability and multidirectional differentiation potential. *In vivo*, MSCs have homing/migration and immunosuppression functions. Several clinical trials are currently ongoing to test the therapeutic efficiency of MSCs in treating severe degenerative diseases, including Crohn’s disease and graft-versus-host disease.

#### 3.2.1 Role of MSC-Derived Exosomes in Cancer Development

Exosomes produced by MSCs contain various kinds of proteins, DNA, and RNA, which regulate the biological behaviors of recipient cells and have a vital role in the pathological and physiological processes of several diseases through intercellular communication. Wei *et al.* demonstrated that MSC-derived exosomal miR-223 could protect neuronal cells from apoptosis via the PTEN-PI3K/Akt signaling pathway, and, in turn, suppress the progression of Alzheimer’s diseases (Wei et al., 2020). In addition, Sun *et al.* revealed the immunomodulatory effects between MSCs-derived exosomes and rheumatoid arthritis (Liu et al., 2020). Recently, some reports indicated a pro- and anti-cancer functional role of MSCs-derived exosomes in cancer. Tao *et al.* (Du et al., 2014) found that exosomes derived from human Wharton’s jelly–derived MSCs (HWJ-MSCs) promote the proliferation and invasion of human renal cancer cells. Krishna *et al.* (Vallabhaneni et al., 2015) suggested that bone marrow MSC–derived exosomes accelerate the growth of MCF-7 breast cancer cells. In addition, Si *et al.* (Shi et al., 2016) showed that human bone marrow MSC–derived exosomes promote the proliferation and metastasis of nasopharyngeal carcinoma. Besides the pro-cancer effects, MSC-derived exosomes also have an anti-cancer role in bladder cancer (Wu et al., 2013), multiple myeloma (Roccaro et al., 2013), ovarian cancer (Reza et al., 2016), and prostate cancer (Takahara et al., 2016). Zhang *et al.* found that bone marrow MSC–derived exosomes may inhibit osteosarcoma cells’ proliferation, invasion, and metastasis by specifically delivering miR-206 (Zhang H. et al., 2020). Furthermore, Jong *et al.* found that mouse bone marrow MSC–derived exosomes reduced vascular endothelial growth factor (VEGF) expression in breast cancer to inhibit angiogenesis in tumor tissues, thereby suppressing tumor growth in mice with breast cancer (Lee et al., 2013).

#### 3.2.2 Application of MSCs-Derived Exosomes as Drug Carriers

**FIGURE 3:**
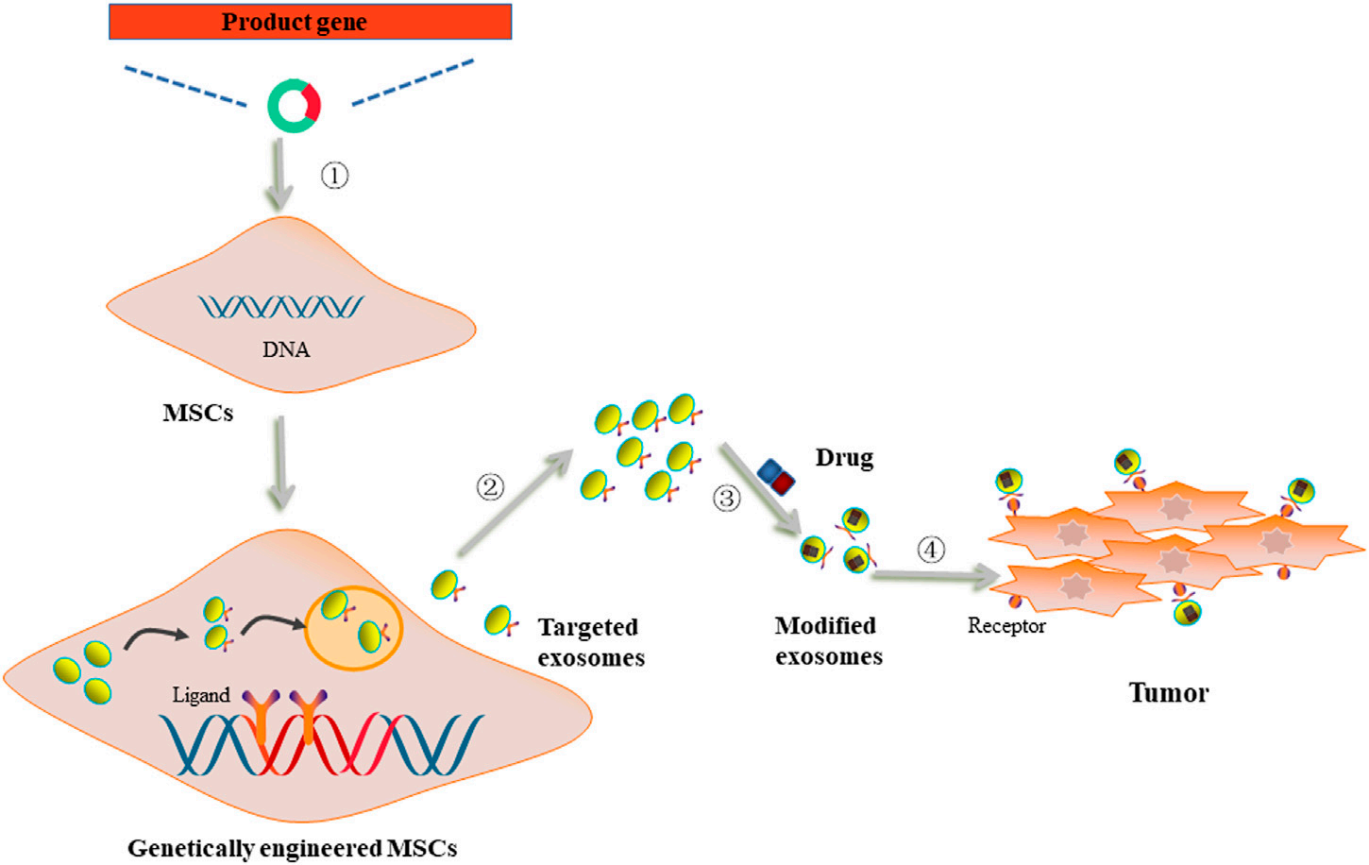
The utilization of genetically engineered MSCs-derived exosomes as drug carriers. ① MSCs were transfected with vectors carrying product genes. ②Genetically engineered MSCs secreted exosomes with acquired targetability to cancer cells. ③ The targeted exosomes were loaded with anti-cancer drugs. ④ Modified exosomes were internalized by cancer cells through ligand/receptor interaction to deliver drugs to exert anti-cancer effects.

Although the function of MSC-derived exosomes in cancer is controversial, these exosomes have been widely studied as drug carriers to exert their anti-cancer effects. Hong *et al.* revealed that exosomes generated from bone marrow-derived MSCs could convey siRNA-GRP78 to recipient liver cancer cells and inhibit the expression of GRP78 so as to enhance the sensitivity of cancer cells to sorafenib (Li H. et al., 2018). Moreover, Brien *et al.* demonstrated that human bone marrow MSCs-derived exosomes carrying miR-379 could significantly decrease cyclooxygenase (COX)-2 expression and suppress the proliferation of breast cancer cells (O'Brien et al., 2018). More recently, several reports suggested that MSCs could be edited using genetic engineering (Figure 3).

Designed ankyrin repeat protein (DARPin) is a small-molecule binding protein with a high affinity to Her 2. Hosna *et al.* demonstrated that engineered MSCs with lysosome-associated membrane glycoprotein 2 (LAMP2)-DARPin could produce exosomes rich in LAMP2-DARPin. After being loaded with DOX, these modified exosomes could selectively target Her 2 positive breast cancer cells and, in turn, exert their cytotoxicity (Gomari et al., 2018). Moreover, Elnaz *et al.* demonstrated that 5TR1 aptamer modified MSCs-derived exosomes carrying DOX could specially bind to Mucin 1 receptor expressed on the surface of colorectal cancer cells and significantly reduce the cytotoxicity of DOX in normal cells (Bagheri et al., 2020).

Despite the abilities of genetically engineered MSCs to endow their derived exosomes with targetability, the pro-cancer effects of these exosomes should not be ignored and need to be further elucidated before clinical application.

### 3.3 Human Embryonic Kidney 293 Cell–Derived Exosomes

Mammalian cells, including HEK293, HT-1080, and NS0 cells, represent important manufacturing platforms in bioengineering and are widely used for the production of recombinant therapeutic proteins and vaccines. Among them, HEK293 cells provide attractive advantages for the development of recombinant proteins or adenovirus productions, such as the human-like post-translational modification of protein molecules to support a better biological activity and high transfection efficiency to produce high-quality recombinant proteins. Recent studies have shown that exosomes derived from HEK293 cells have great potential to deliver anti-cancer drugs.

#### 3.3.1 Role of HEK293 Cell-Derived Exosomes as Drug Carriers

Yao *et al.* demonstrated that HEK293 cell-derived exosomes carrying miR-204-5p could decrease the expression of miR-204-5p targeted genes RAB22A and Bcl-2 and inhibit proliferation of colorectal cancer, breast cancer, lung cancer, gastric cancer, and glioma (Yao et al., 2020).

The hepatocyte growth factor (HGF) could promote tumor progression by activating downstream pathways, such as phosphatidylinositol 3-kinase-AKT, RAS-mitogen-activated protein kinase, and vascular endothelial growth factor (VEGF) signaling (Finisguerra et al., 2016). Zhang *et al.* showed that HEK293 cell-derived exosomes could carry siRNA-HGF to gastric cancer cells and inhibit cell proliferation by decreasing VEGF expression (Zhang et al., 2018).

The overexpression of cellular-mesenchymal epithelial transition (c-MET) is one of the mechanisms of cisplatin resistance in gastric cancer. Zhang *et al.* found that HEK293 cell-derived exosomes transferring siRNA-c-MET could significantly inhibit the expression level of c-MET and reverse cisplatin resistance of gastric cancer (Zhang Q. et al., 2020).

#### 3.3.2 Genetically Engineered HEK293 Cell-Derived Exosomes as Drug Carriers

Similar to MSCs, HEK293 cells could also be genetically engineered to endow their derived exosomes with targetability. Limoni *et al.* infected HEK293 cells with LAMP-DARPin chimeric gene and obtained their derived exosomes. Consequently, exosomes carrying siRNA-TPD52 could target Her 2 positive breast cancer cells and obviously suppress oncogene TPD52 expression (Limoni et al., 2019). Furthermore, Daniele *et al.* developed HEK293 cells with Lamp2b-IL3 to acquire their derived exosomes; exosomes loaded with siRNA-BCR-ABL could specifically target IL3 receptor-positive chronic myeloid leukemia cells and exert obvious anti-cancer effects (Bellavia et al., 2017). In addition, Bai *et al.* transfected HEK293T cells with Lamp2b-tLyp-1 plasmid and isolated their derived exosomes, which could specifically target toneuropilin (NRP) overexpressing lung cancer stem cells and significantly decrease stemness-associated SOX expression to attenuate stemness phenotypic features of these cells via delivering siRNA (Bai et al., 2020). Moreover, Liang *et al.* transfected HEK293 cells with Target-Her 2-Lamp2-GFP (THLG) and purified their derived exosomes, which when loaded with anti-cancer drug 5-Fluorouracil (5-Fu) could directly target Her 2 positive colon cancer cells and inhibit the growth of colon cancer in mice (Liang et al., 2020).

### 3.4 Dendritic Cell-Derived Exosomes

DCs originate from bone marrow pluripotent hematopoietic stem cells and are one of the most potent antigen-presenting cells. These cells can activate resting T cells and promote antigen presentation via major histocompatibility complex (MHC) molecules; thus, they have an important role in initiating, regulating, and maintaining the immune responses as well as participating in the immunotherapy of cancer.

#### 3.4.1 Role of DC-Derived Exosomes in Immunotherapy of Cancer

Recent studies revealed that DC-derived exosomes carry MHC molecules on their surfaces and own antigen-presenting ability, suggesting that DC-derived exosomes participate in immune regulation (Pitt et al., 2014). Than *et al.* demonstrated that exosomes isolated from cryopreserved umbilical cord blood mononuclear cell-derived DCs (cryo CBMDCs) and pre-treated with lung cancer cell lysates could induce proliferation of allogeneic T-cells to exert greater cytotoxic activity against lung cancer cells compared with untreated cryo CBMDCs-derived exosomes (Than et al., 2020). Martina *et al.* found that after co-incubating bone marrow-derived DCs with melanoma cell lysates and poly (I:C), derived exosomes could significantly promote proliferation of CD8^+^ and CD4^+^ T cells, thus enhancing the cytotoxicity of T cells to melanoma cells (Damo et al., 2015). In addition, Chen *et al.* revealed that derived exosomes, obtained after treating DCs with poly (I:C) and E7_49-57_ peptide, could promote cytotoxic activity and interferon (IFN)-γ excretion of CD8^+^ T cells, thereby significantly inhibiting the growth of cervical cancer (Chen S. et al., 2018). Recently, researchers observed that exosomes derived from a recombinant adeno-associated viral vector (rAAV)-carrying alpha-fetoprotein (AFP) gene (rAAV/AFP)-transfected DCs could effectively stimulate the proliferation of naive T cells and induce the activation of cytotoxic T lymphocytes (CTLs), thereby exhibiting immune responses against hepatocellular carcinoma (Li J. et al., 2018). Similarly, Zhen *et al.* demonstrated that AFP-rich DC-derived exosomes could elicit potent antigen-specific immune responses, significantly suppressing hepatocellular carcinoma growth and prolonging the survival of tumor-bearing mice (Lu et al., 2017). Phung *et al.* clarified that exosomes secreted by chicken egg albumin (OVA)-treated DCs could greatly activate T cells, stimulate cell proliferation, and increase the CTL/Treg ratio in tumor tissues, thus resulting in an obvious inhibition of tumor growth in melanoma-bearing mice (Phung et al., 2020).

#### 3.4.2 Role of DC-Derived Exosomes as Drug Carriers

**FIGURE 4:**
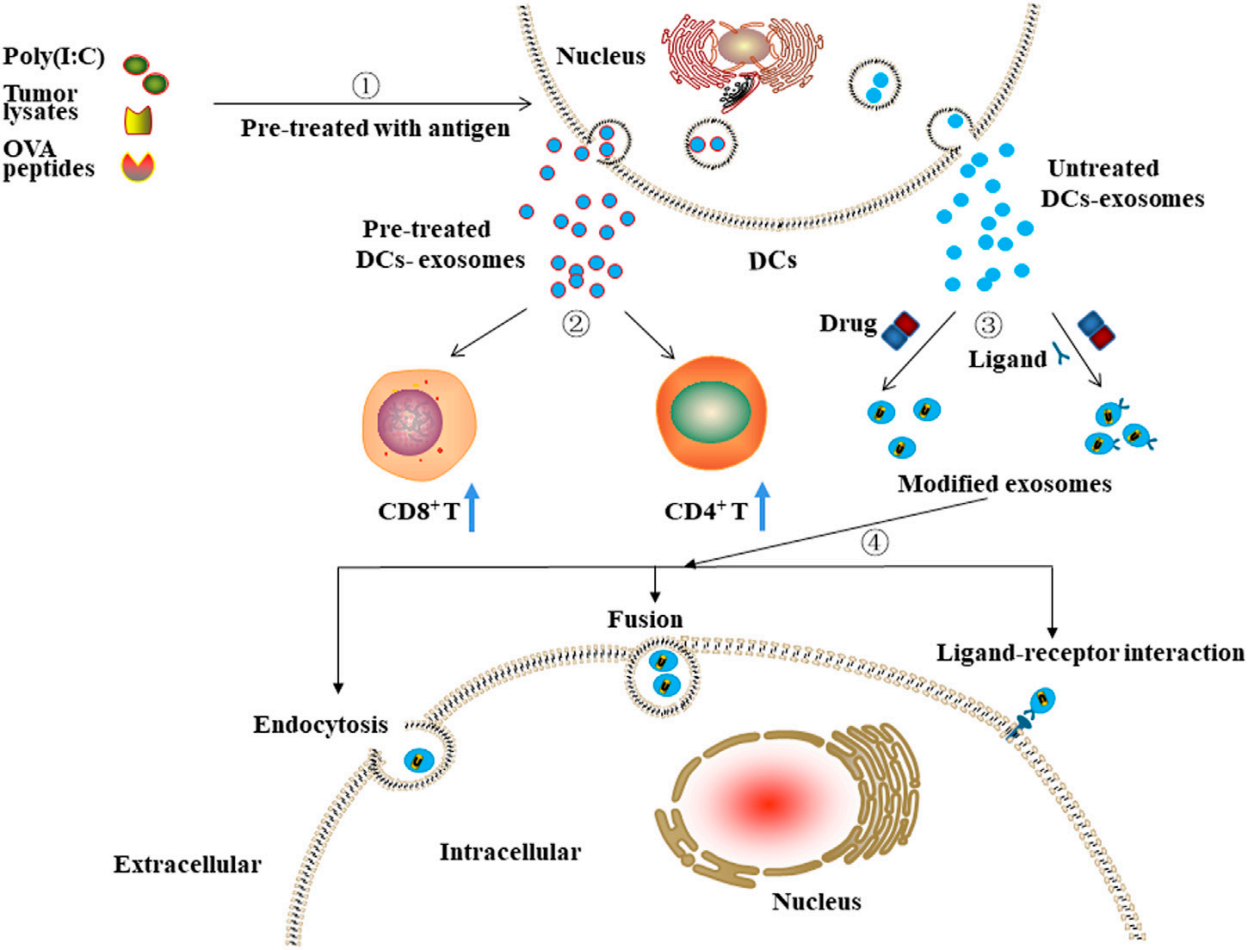
The potential clinical application of DCs-derived exosomes.①-②Exosomes isolated from pre-treated DCs can stimulate the proliferation of immune cells, including CD8^+^ and CD4^+^ T cells. ③-④ Exosomes isolated from untreated DCs and then modified with targeting moieties or/and loaded with anti-cancer drugs can exert anti-cancer effects through ligand-receptor interaction, fusion, or endocytosis.

DC-derived exosomes may also be used as carriers of anti-cancer drugs (Figure 4). Man *et al.* showed that DC-derived exosomes loaded with 5-Fu could significantly suppress the tumor growth of colon cancer in mice compared to the anti-cancer effects of free 5-Fu (Xu et al., 2020). Moreover, Zou *et al.* reported that aptamer sgc8, which specifically recognizes membrane-expressed protein tyrosine kinase 7 (PTK7), could be modified onto the surface of DC-derived exosomes. After being loaded with DOX, these exosomes could efficiently deliver DOX to cancer cells overexpressing PTK7 and thus exert their anti-cancer effects (Zou et al., 2019). Moreover, Tian *et al.* suggested that exosomes derived from Lamp2b-iRGD plasmid-transfected DCs and then loaded with DOX could inhibit the proliferation of breast cancer cells without affecting other (healthy) cells (Tian et al., 2014). Similarly, Xin *et al.* reported that iRGD-modified DC-derived exosomes loaded with recombinant methioninase (rMETase) could be specifically delivered to tumor tissues and exert significantly anti-cancer effects *in vivo* (Xin et al., 2020).

DC-derived exosomes as drug carriers can improve the immunosuppressive microenvironment of tumor tissues through activation and increasing proportion of proliferating T cells and can exert cytotoxicity by delivering anti-cancer drugs, thus are considered to have great potential in the treatment of cancer.

### 3.5 Cancer Cell-Derived Exosomes

#### 3.5.1 Role of Cancer Cell-Derived Exosomes as Drug Carriers

Recent studies found that cancer cell-derived exosomes share some integrins with cancer cells, which exhibit a natural targeting effect known as the “Trojan horse” property. Due to this special characteristic, cancer cell-derived exosomes might be considered a suitable drug delivery system in cancer therapy. Liu *et al.* reported that ovarian cancer cell-derived exosomes loaded with triptolide could accumulate in tumor tissues and inhibit ovarian cancer in mice (Liu et al., 2019). Li *et al.* demonstrated that fibrosarcoma cell HT1080–derived exosomes loaded with DOX showed a better anti-cancer effect through enhancing therapeutic retention in tumor tissues and significantly reducing DOX-induced cardiac toxicity, compared to free DOX (Qiao et al., 2020). In addition, Fu *et al.* transfected tripartite motif-containing 3 (TRIM3) plasmid into gastric cancer cells to acquire TRIM3-overexpressing exosomes and found that these exosomes could downregulate the expression levels of OCT4, SOX2, and N-cadherin of gastric cancer cells, thereby inhibiting the tumor growth in mice (Fu et al., 2018). Nie *et al.* further demonstrated that breast cancer cell-derived exosomes carrying integrin beta 4 (ITGβ4) exhibited obvious targetability to surfactant protein C overexpressing lung cancer cells. After being loaded with miRNA-126, these exosomes showed an obvious suppression of tumor growth in lung cancer-bearing mice (Nie et al., 2020).

#### 3.5.2 Shortcomings of Cancer Cell-Derived Exosomes as Drug Carriers

Despite the innate targetability of cancer cell-derived exosomes, the pro-cancer effects of these exosomes made it far away from clinical application. Chen *et al.* found that highly metastatic hepatocellular carcinoma cell-derived exosomes could communicate with low metastatic hepatocellular carcinoma cells to increase their migration, chemotaxis, and invasion by epithelial-mesenchymal transition through MAPK/ERK pathway (Chen L. et al., 2018). Moreover, Akihiko *et al.* found that exosomes derived from ovarian cancer could contribute to ovarian cancer cells invasion via transferring miR-99a-5p, which further up-regulates the expression of fibronectin and vitronectin (Yoshimura et al., 2018). Likewise, the cancer-promoting activity of cancer cell-derived exosomes has also been verified in melanoma, colorectal, and prostate cancer (Saari et al., 2015; Felicetti et al., 2016; Zeng et al., 2018).

### 3.6 Erythrocyte-Derived Exosomes

During the differentiation of erythroid cells, a vast program of maturation occurs. Reticulocyte maturation into erythrocytes is the final step of erythropoiesis that occurs in blood circulation. At that time, the intracellular organelles, including the nucleus, mitochondria, ribosomes, lysosomes, endoplasmic reticulum, and Golgi apparatus, are decayed or eliminated, and the typical cellular biconcave is formed. Recently, some researchers found that, due to the deficiency of nuclear and mitochondrial DNA, erythrocytes-derived exosomes could be considered as a promising drug delivery system for anti-cancer therapy. Qi *et al.* found that reticulocyte-derived exosomes combined with superparamagnetic nanoparticle clusters and then loaded with DOX could target breast cancer or hepatocellular carcinoma and significantly exert anti-cancer effects *in vitro* and *in vivo* under an external magnetic field (Qi et al., 2016). Antisense oligonucleotides (ASOs) and CRISPR–Cas9 genome editing guide RNAs (gRNAs) are RNA therapeutics, which could inhibit tumor progression by regulating the levels of specific genes involved in cell proliferation, differentiation, and survival, with the characteristics of high specificity and low cytotoxicity. Based on this, Waqas *et al.* demonstrated that erythrocytes-derived exosomes loaded with ASOs and Cas9mRNA could inhibit the proliferation of acute myeloid leukemia and breast cancer cells *in vitro* and *in vivo* (Usman et al., 2018). Recently, Zhan *et al.* demonstrated that erythrocytes-derived exosomes modified with phosphatidylcholine and then loaded with DOX could decrease the proliferation of glioma cells via enhanced cancer cell uptake and improve intracellular DOX delivery efficiency (Zhan et al., 2021).

## 4 Challenges and Prospects in Using Exosomes as Drug Carriers

Over recent years, many reports suggested using exosomes as a suitable drug delivery system for cancer therapy. Compared with liposomes and nanoparticles, exosomes offer high biocompatibility, low immunogenicity, the ability to penetrate biological barriers easily, and non-toxic accumulation. Therefore, developing a drug delivery system based on exosomes is of great scientific significance and has potential clinical application prospects. However, the clinical application of exosomes as drug carriers still faces some challenges.

**FIGURE 5:**
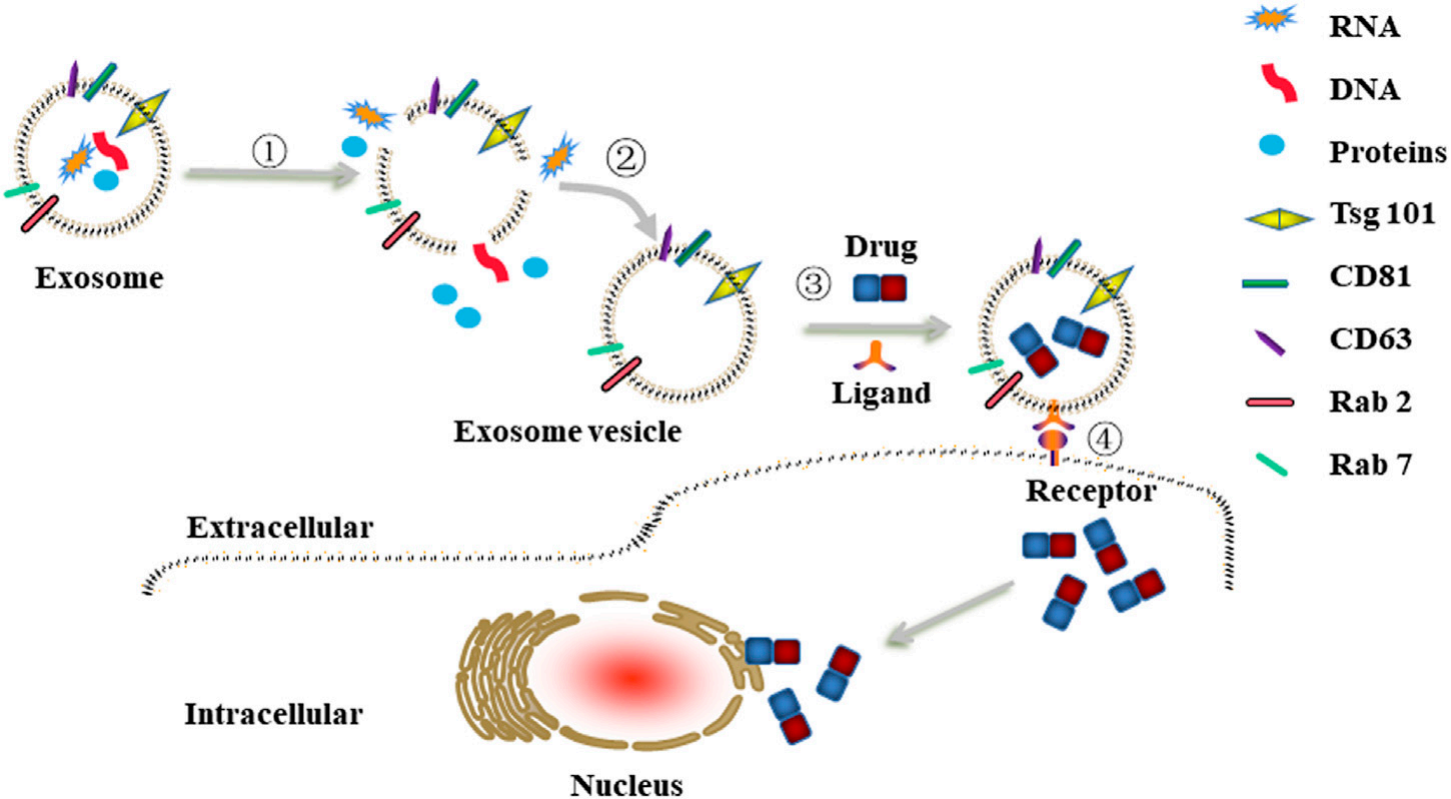
Schematic illustrations of engineered exosomes as drug carriers. ①-② The removal procedure of natural contents in cells-derived exosomes to construct exosome vesicle. ③ Exosome vesicle was modified with targeting moieties (e.g., folic acid, hyaluronan) via co-incubation and then loaded with anti-cancer drugs (e.g., DOX, PTX, 5-Fu) via electroporation or sonication to obtain engineered exosomes. ④The engineered exosomes were internalized by cancer cells through ligand/receptor interaction.

Besides cell heterogeneity, exosomes may be different in size, have different content and functional abilities, which raises the questions about their safety. Therefore, before moving forward with the clinical work, it is necessary to discharge the natural contents of exosomes to avoid the pro-cancer effects and then modify them with targeting moieties or/and load them with anti-cancer drugs to obtain engineered exosomes (Figure 5). Recently, Li *et al.* revealed that use of hypotonic treatment to remove the contents of macrophage-derived exosomes was feasible. After that, these exosomes loaded with anti-cancer drugs could also exert anti-cancer effects against breast cancer (Li S. et al., 2020). Based on this, further experiments should be performed to extend the applicability of hypotonic treatment in other cells-derived exosomes so as to avoid the heterogeneities of exosomes, which could increase the security risk of exosomes as drug carriers.

The yield of exosomes may vary in accordance with the type of donor cells. Tian *et al.* revealed that the yield of immature dendritic cells (iDC)-derived exosomes is very low (Yeo et al., 2013; Tian et al., 2014). Moreover, Wang *et al.* demonstrated that breast cancer cells produce fewer exosomes compared with normal cells in the same number per unit time (Chiu et al., 2016). Compared to other exosomes, milk-derived exosomes offered a higher yield; however, acquiring targetability to cancer through genetic engineering of milk-derived exosomes was very difficult. Moreover, milk-derived exosomes were currently restricted to the modification of hyaluronan or folic acid, which were not targeting cancer cells especially. Therefore, genetically engineered milk-derived exosomes or the strategies to increase the yield of exosomes should be further explored. Recently, some researchers proposed that the yield of exosomes might depend on the different functions of the endosomal sorting complex (Rab protein and soluble N-ethylmaleimide-sensitive factor attachment protein receptor (SNARE) protein family), which regulates the process of exosomal synthesis and secretion from host cells. Therefore, more in-depth investigations into functional roles of endosomal sorting complex in host cells should be carried out in the future.

At present, many methods have been used to load anti-cancer drugs into exosomes, including co-incubation, electroporation, and chemical transfection (Table 1). However, the drug loading efficiency when using different methods may vary. Farrukh *et al.* compared the loading efficiency of milk-derived exosomes with siRNA between chemical transfection and electroporation and found that the loading efficiency of siRNA into exosomes via chemical transfection was 30% while it was 35% through electroporation (Aqil et al., 2019). Kim *et al.* discovered that the loading efficiency of macrophages-derived exosomes with PTX when using co-incubation, electroporation, and sonication is different; the loading efficiency of sonication was 28.29%, while co-incubation and electroporation were 1.44 and 5.3%, respectively (Kim et al., 2016). Moreover, the loading efficiency of exosomes with anti-cancer drugs also depends on the types of host cells. Thakur *et al.* demonstrated that glioblastoma stem cell or glioma cell-derived exosomes loaded with DOX in the same condition exhibited different loading efficiency with 19.7 and 7.86%, respectively (Thakur et al., 2020). Therefore, ways to optimize the above methods with the greatest drug loading efficiency need to be urgently explored.

**TABLE 1:** The summary of exosomes as drug carriers for cancer therapy.

Source of exosomes	Loading drugs	Loading method	Cancer type	Type	Study outcome	References
Milk	withaferin A	Incubation	A549 cells	Vivo	Enhance tumor reduction	Munagala et al. (2016)
Milk	celastrol	Incubation	A549 cells	Vivo	Enhance antitumor efficacy	Aqil et al. (2016)
Milk	Anthos	Incubation	A2780 cells	Vivo	Inhibit t tumor growth	Aqil et al. (2017)
Milk	si-KRAS^G12S^	Transfection	A549 cells	Vivo	Suppress tumor growth	Aqil et al. (2019)
Milk	Bcl-2-siRNA	Ultrasonic	Panc28 cells	Vivo	Inhibit tumor growth	Tao et al. (2020)
Milk	DOX	Incubation	MDA-MB-231, MCF-7 cells and A549 cells	vitro	Trigger tumor cells death	Li D. et al. (2020)
Milk	PTX	Incubation	A549 cells	Vivo	Enhance anti-tumor efficiency	Kandimalla et al. (2021)
BM-MSC	siRNA-GRP78	Transfection	HepG2 and PLC-SR cells	Vivo	Inhibit tumor growth and overcome drug resistance	Li H. et al. (2018)
BM-MSCs	miR-379	Transfection	T47D and HCC cells	Vivo	Inhibit tumor growth	O'Brien et al. (2018)
MSCs	DOX	Electroporation	MDA-MB-231 and BT-474 cells	Vitro	Increase the target ability to Her2(+)breast cancer	Gomari et al. (2018)
BM-MSCs	DOX	Electroporation	C26 tumor cells	Vivo	Suppress tumor growth	Bagheri et al. (2020)
HEK293	miR-204-5p	Transfection	HCT116 cells	Vivo	Inhibit tumor growth	Yao et al. (2020)
HEK293	HGF siRNA	Transfection	SGC-7901 gastric cells	Vivo	Decrease the growth rates of tumors and blood vessels	Zhang et al. (2018)
HEK293	c-Met siRNA	Transfection	SGC7901/DDP gastric cells	Vivo	Revers chemotherapy resistance and inhibit tumor growth vivo	Zhang Q. et al. (2020)
HEK293	siRNA-TPD52	Transfection	SKBR3 cells	Vitro	Down-regulate TPD52 gene expression	Limoni et al. (2019)
HEK293	BCR-ABL-siRNA or imatinib	Transfection	LAMA84 cells and K562R cells	Vivo	Reduce the tumor size	Bellavia et al. (2017)
HEK293	siRNA	Transfection	A549 cells	Vitro	Reduce the stemness of cancer stem cells	Bai et al. (2020)
HEK293	miR-21i and 5-FU	Transfection	HCT-1165FR	Vivo	Inhibit tumor growth and reverse drug resistance	Liang et al. (2020)
DCs	5-FU	Electroporation	CT26 cells	Vivo	Inhibit tumor growth	Xu et al. (2020)
imDCs	DOX	Electroporation	CEM cells	Vitro	Enhanced cellular accumulation of DOX	Zou et al. (2019)
imDCs	DOX	Electroporation	MDA-MB-231 cells	Vivo	Inhibit tumor growth vivo	Tian et al. (2014)
imDCs	rMETase	Transfection	SGC7901 cells	Vivo	Inhibit tumor growth	Xin et al. (2020)
SKOV3 cells	triptolide	Sonication	SKOV3 cells	Vivo	Inhibit tumor growth and proliferation	Liu et al. (2019)
HT1080 cells	DOX	Membrane extrusion	HT1080 cells	Vivo	Inhibit tumor growth	Qiao et al. (2020)
MGC-803	TRIM3	Transfection	MGC-803 cells	Vivo	Suppress cancer growth and metastasis	Fu et al. (2018)
MDA-MB-231 cells	miRNA-126	Transfection	A549 cells	Vivo	Inhibit the formulation of lung metastasis	Nie et al. (2020)
Reticulocytes	DOX	Incubation	Hepatoma 22 cells	Vivo	Inhibit tumor growth and proliferation	Qi et al. (2016)
RBCs	ASOs and Cas9mRNA	Transfection	MOLM13, NOMO1 and CA1a cells	Vivo	Inhibit tumor growth	Usman et al. (2018)
RBCs	DOX、anti-miR21	Incubation	Glioma	Vitro	Inhibit proliferation of tumor cells	Zhan et al. (2021)
SF7761 and U251-GMs	DOX	Microfluidics	SF7761 and U251-GMs		Inhibit the proliferation of glioma	Thakur et al. (2020)

## 5 Conclusion

Although there are significant challenges and difficulties in applying exosome-based drug delivery systems, this natural vesicle has great potential in the biomedical field, especially in anti-cancer therapy. Yet, certain problems need to be solved before they can be used in clinical practice. Thus, the scientific community should further focus on the exploration of these problems.
